# Modified Citrus Pectin-Mediated Gal-3 Reduction Attenuates Metabolic Dysfunction-Associated Steatohepatitis in an Experimental Model

**DOI:** 10.3390/cells15171544

**Published:** 2026-08-27

**Authors:** Anne R. M. Santos, Alessandra G. Cruz, Felipe N. Camargo, Jessica D. M. Santos, Jorge F. A. Model, José F. T. Silva, Sandro L. Matos, Layanne C. C. Araujo, João Paulo Camporez

**Affiliations:** 1 Department of Physiology, Ribeirao Preto School of Medicine, University of Sao Paulo, Ribeirao Preto 14049-900, Brazil; annemello1928@hotmail.com (A.R.M.S.); alessandracruz@usp.br (A.G.C.); felipe_camargo@usp.br (F.N.C.); jessicadenielle@usp.br (J.D.M.S.); joseturino@usp.br (J.F.T.S.); sandroleaomatos@usp.br (S.L.M.); 2 Superior Institute of Biomedical Sciences, Faculty of Education and Integrated Sciences, State University of Ceara, Fortaleza 60714-903, Brazil

**Keywords:** Galectin-3, modified citrus pectin, metabolic dysfunction-associated steatohepatitis, liver fibrosis, inflammation, hepatic insulin resistance, hepatic steatosis

## Abstract

Metabolic dysfunction-associated steatohepatitis (MASH) is characterized by hepatic steatosis, inflammation, fibrosis, and metabolic dysfunction, with limited therapeutic options currently available. This study investigated whether pharmacological inhibition of Galectin-3 (Gal-3), a β-galactoside-binding lectin implicated in inflammation and fibrosis, using modified citrus pectin (MCP), attenuates established MASH in ApoE-deficient mice. Mice were assigned to four groups: standard chow (CTL), standard chow plus MCP (CTL + MCP), Western diet with fructose-enriched drinking water (WD), and WD plus MCP (WD + MCP). MCP (1% in drinking water) was administered during the final four weeks of the 11-week experimental protocol, after the establishment of metabolic dysfunction and hepatic steatosis. Compared with untreated WD mice, WD + MCP mice exhibited improved glucose tolerance, as evidenced by a 22.9% reduction in glucose area under the curve (*p* = 0.01), and enhanced hepatic insulin signaling, reflected by a 254.1% increase in insulin-stimulated AKT2 phosphorylation (*p* = 0.0002). MCP treatment also reduced hepatic lipid accumulation (39.8%, *p* = 0.0055) and hepatic triglyceride content (26.1%, *p* = 0.0003), accompanied by reductions in circulating triglyceride and cholesterol concentrations. Furthermore, WD + MCP mice exhibited reduced hepatic Gal-3 expression (25.8%, *p* = 0.0199), decreased F4/80 immunoreactivity (49.2%, *p* = 0.0061), attenuated JNK phosphorylation (46.6%, *p* = 0.0118), reduced collagen deposition (47.0%, *p* = 0.0002), and lower plasma ALT (52.9%, *p* = 0.0009) and AST (48.8%, *p* = 0.04) activities. Collectively, these findings demonstrate that MCP attenuates multiple metabolic, inflammatory, fibrotic, and hepatic injury features of established Western diet-induced MASH, supporting Gal-3 as a promising therapeutic target and MCP as a potential pharmacological strategy for MASH.

## 1. Introduction

Metabolic dysfunction-associated steatohepatitis (MASH) is a leading cause of chronic liver disease and represents a growing global health burden. It develops from metabolic dysfunction-associated steatotic liver disease (MASLD), which affects approximately one-third of the adult population worldwide and is projected to increase substantially over the coming decades [1,2,3,4]. Driven by the rising prevalence of obesity, type 2 diabetes mellitus, and other cardiometabolic disorders, MASH has become a major therapeutic challenge, as effective pharmacological strategies to halt or reverse disease progression remain limited [2].

MASH progression is driven by metabolic dysfunction and chronic inflammation that culminate in hepatocellular injury. Excess nutrient availability promotes ectopic hepatic lipid accumulation and insulin resistance, mediated in part by bioactive lipid intermediates such as diacylglycerol (DAG) [5,6]. Adipose tissue dysfunction further amplifies this process by promoting macrophage recruitment and the release of pro-inflammatory cytokines and free fatty acids, thereby sustaining hepatic inflammation and systemic insulin resistance [7,8]. In parallel, activation of stress-responsive pathways, including c-Jun N-terminal kinase (JNK), directly contributes to hepatocellular injury [9].

Histologically, MASH is characterized by steatosis, lobular inflammation, hepatocellular ballooning, and progressive fibrosis, all of which are associated with an increased risk of cardiovascular, renal, and metabolic complications [10,11].

Hepatic fibrosis is the strongest predictor of liver-related and overall mortality in MASH [12,13]. Persistent inflammation drives extracellular matrix (ECM) remodeling by activating hepatic stellate cells (HSCs), which transdifferentiate into collagen-producing myofibroblasts [14]. Progressive matrix deposition promotes sinusoidal capillarization—characterized by the loss of endothelial fenestrations and hepatocyte microvilli—thereby impairing metabolic and signaling exchanges between the portal circulation and hepatocytes [15]. Furthermore, transforming growth factor-β (TGF-β) signaling maintains chronic HSC activation, accelerating ECM deposition and fibrosis [16,17].

Galectin-3 (Gal-3) has emerged as a central regulator of the inflammatory and fibrotic responses underlying MASH progression. As a chimeric β-galactoside-binding lectin, Gal-3 recognizes glycoconjugates through its carbohydrate recognition domain (CRD) to regulate immune responses, tissue remodeling, and fibrogenesis [16,17,18]. Predominantly expressed by macrophages and activated fibroblasts, Gal-3 promotes HSC activation and potentiates TGF-β-dependent fibrotic signaling [19,20,21,22]. Consistent with these functions, Gal-3 expression is markedly elevated in both MASLD/MASH patients and experimental disease models, underscoring its central role in pathogenesis [23,24,25]. Collectively, these findings highlight Gal-3 as a promising therapeutic target for mitigating hepatic inflammation and fibrosis.

Modified citrus pectin (MCP) is one of the best-characterized pharmacological inhibitors of Gal-3. Chemical and structural modifications reduce its molecular weight and increase its bioavailability, enabling efficient gastrointestinal absorption and systemic interaction with Gal-3 [26,27]. Beyond direct Gal-3 inhibition, MCP may also influence intestinal physiology and nutrient metabolism, providing an additional axis for modulating liver function [28,29]. Experimental studies have demonstrated the anti-inflammatory and anti-fibrotic effects of MCP across cardiac, hepatic, and renal disease models, primarily through the suppression of Gal-3-mediated signaling [30,31].

Despite these promising findings, the therapeutic potential of MCP in established MASH remains poorly defined. Specifically, whether oral administration of MCP can reverse the histopathological and metabolic abnormalities of advanced disease has yet to be determined. Therefore, we evaluated the therapeutic effects of oral MCP administration in a Western diet-fed, ApoE-deficient mouse model of MASH, testing the hypothesis that Gal-3 inhibition attenuates hepatic injury, fibrosis, and metabolic dysfunction.

## 2. Materials and Methods

### 2.1. Animals

Male apolipoprotein E-deficient (ApoE−/−, C57BL/6J background, The Jackson Laboratory, RRID:IMSR_JAX:002052) mice were housed under controlled temperature (22 ± 2 °C) and a 12 h light/dark cycle, with ad libitum access to food and water. All experimental procedures were conducted in accordance with the Guidelines for the Ethical Use of Animals in Applied Animal Behavior and Welfare Research and were approved by the Animal Care and Use Committee of the Ribeirão Preto Medical School, University of São Paulo (FMRP-USP) (CEUA 1376/2024).

### 2.2. Experimental Design and Treatment

**Figure 1:**
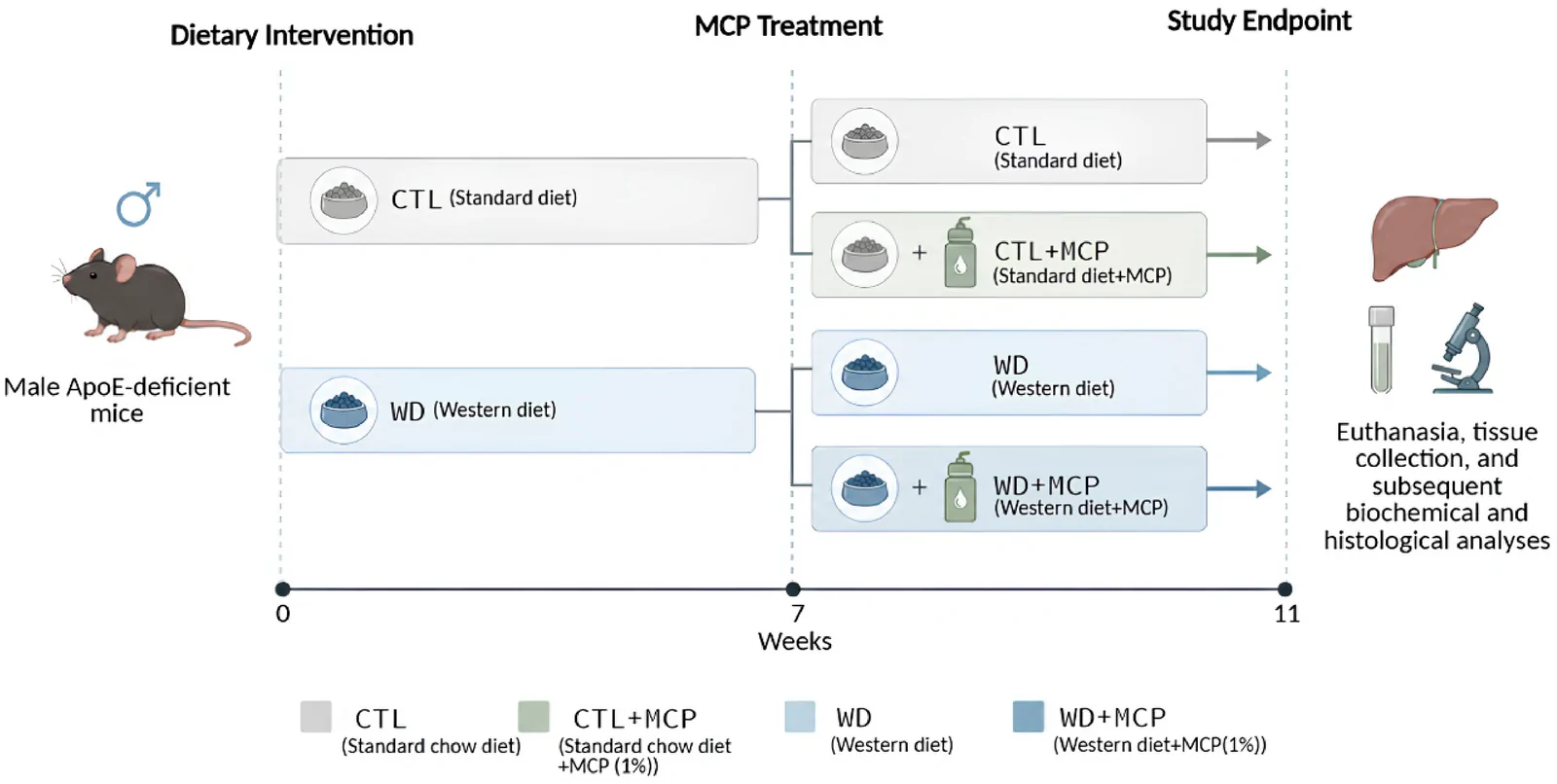
Experimental design. Male ApoE-deficient mice were randomly assigned to receive either a standard chow diet (CTL) or a Western diet (WD) for 7 weeks. Subsequently, each dietary group was subdivided to receive either vehicle or 1% modified citrus pectin (MCP) in the drinking water for an additional 4 weeks, resulting in four experimental groups: CTL, CTL + MCP, WD, and WD + MCP. At the end of the 11-week experimental period, mice were euthanized, and blood and tissue samples were collected for biochemical, molecular, and histological analyses.

Mice were fed either a standard chow diet (CTL; Nuvilab^®^ CR-1; 3.5% fat, 19% protein, 56% carbohydrate; 3.2 kcal/g, Nuvilab, Seoul, Republic of Korea) or a Western diet (WD; Research Diets D12079B; 40% fat, 17% protein, 43% carbohydrate, supplemented with 0.15% cholesterol; 4.7 kcal/g), as shown in Table 1. At the beginning of the experimental protocol, animals were randomly assigned to four groups: CTL, CTL + MCP, WD, and WD + MCP. The CTL and WD groups remained on their respective diets throughout the study, whereas the CTL + MCP and WD + MCP groups received 1% modified citrus pectin (MCP) in the drinking water during the last four weeks of the protocol [30,32], following seven weeks of dietary intervention and establishment of MASH [24], which represents an ingestion of approximately 2 g/kg/day of MCP. Mice fed the Western diet also received 10% fructose in the drinking water throughout the experimental period (Figure 1). Depending on the analysis performed, each experimental group consisted of 5–9 animals. Body weight, food intake, and water intake were weighed weekly, with food and water consumption measured per cage and normalized to the number of animals.

### 2.3. Body Composition

Body composition was assessed before and at the end of the experimental protocol by nuclear magnetic resonance using a Minispec LF50 analyzer (Bruker, Billerica, MA, USA). Body weight, lean mass, fat mass, and body fat percentage were determined.

### 2.4. Glucose Tolerance Test and Hepatic Insulin Sensitivity

Glucose homeostasis was evaluated by an intraperitoneal (i.p.) glucose tolerance test (GTT) following a 6 h fast. Mice received glucose (2 g/kg body weight; 20% dextrose solution, i.p.), and blood samples were collected from the tail vein at 0, 15, 30, 45, 60, 90, and 120 min for blood glucose determination. At baseline, 10 μL of plasma was collected for fasting insulin measurement using a commercial ELISA kit (Mercodia Mouse Insulin ELISA, 10-1247-01; Mercodia AB, Uppsala, Sweden). HOMA-IR and HOMA-β indices were calculated according to Matthews et al. (1985) (HOMA-IR = fasting insulin (µU/L) × fasting glucose (mg/dL)/405) (HOMA-β = 20 × fasting insulin (µU/L)/(fasting glucose (mg/dL) × 0.0555) − 3.5) [33]. Hepatic insulin sensitivity was assessed by acute insulin stimulation through the inferior vena cava. Liver samples were collected immediately before (0 s) and 30 s after insulin administration (from the same animal), snap-frozen in liquid nitrogen, stored at −80 °C, and subsequently analyzed for insulin signaling by Western blot.

### 2.5. Tissue Collection

After a 6 h fast, mice were anesthetized with isoflurane and euthanized for tissue collection. Blood was collected from the inferior vena cava using heparinized syringes, centrifuged at 12,000 rpm (15,294 rcf) for 2 min to obtain plasma, and then stored at −80 °C until biochemical analyses. Liver samples were collected for molecular, biochemical, and histological analyses. Samples designated for molecular and biochemical analyses were snap-frozen in liquid nitrogen and stored at −80 °C, whereas fragments from the left lateral liver lobe were fixed in 10% formaldehyde for histological evaluation.

### 2.6. Plasma Biochemical Analyses

Plasma triglycerides, total cholesterol, alanine aminotransferase (ALT), and aspartate aminotransferase (AST) levels were determined using commercial enzymatic colorimetric assay kits (Bioclin^®^, Quibasa Química Básica Ltda., Belo Horizonte, MG, Brazil).

### 2.7. Hepatic and Fecal Triglyceride Content

Hepatic triglyceride content was determined following lipid extraction using the Bligh and Dyer method [34]. Briefly, liver samples (50–100 mg) were homogenized in chloroform–methanol (2:1, *v*/*v*), then 1 M sulfuric acid was added, and the mixture was centrifuged at 2000 rpm (425 rcf) for 10 min to promote phase separation. The lower organic phase was collected, evaporated overnight, and resuspended in 1 mL of isopropanol. The triglyceride concentration was then determined using a commercial enzymatic colorimetric assay kit (Bioclin, Quibasa Química Básica Ltda., Belo Horizonte, MG, Brazil). Fecal triglyceride content was determined from approximately 100 mg of powdered dry feces, which was homogenized in methanol–chloroform (1:2, *v*/*v*) using an Ultra-Turrax homogenizer (IKA^®^, Staufen, Germany). Samples were centrifuged at 2000 rpm for 10 min, and the liquid phase above the fecal pellet was transferred to a new tube. Subsequently, 0.9% saline was added, the samples were vortexed and centrifuged again at 2000 rpm (425 rcf) for 10 min to promote phase separation. The lower organic phase was collected, air-dried overnight in a fume hood at room temperature, resuspended in 300 μL of ethanol, and triglyceride concentration was determined using a commercial enzymatic colorimetric assay kit (Bioclin^®^, Quibasa Química Básica Ltda., Belo Horizonte, MG, Brazil).

### 2.8. Oral Lipid Tolerance Test

An oral lipid tolerance test (OLTT) was performed after an overnight fast. Mice received 400 μL of lard by oral gavage, and blood samples were collected from the tail vein before lipid administration (0 h) and at 1, 2, 3, and 4 h thereafter. Plasma triglyceride concentrations were measured using a commercial enzymatic colorimetric assay kit (Bioclin^®^, Quibasa Química Básica Ltda., Belo Horizonte, MG, Brazil). Lipid tolerance was evaluated by monitoring plasma triglyceride concentrations over time and calculating the area under the curve (AUC).

### 2.9. Hematoxylin and Eosin (H&E) Staining

Liver samples were fixed in 10% neutral-buffered formalin, while each intestinal segment (duodenum, jejunum, ileum, and colon) was individually arranged using the Swiss-roll technique before fixation. All samples were paraffin-embedded, sectioned at 5 μm, and stained with hematoxylin and eosin (H&E). Liver morphology was evaluated from five images per animal acquired using a Nikon Eclipse Ti-U microscope coupled to a Nikon DS-Ri1 digital camera (20× objective) (Tokyo, Japan). Intestinal morphology was evaluated from 20 images per animal acquired using a 10× objective. Villus length was measured in the duodenum, jejunum, and ileum from the base to the tip of the villi, whereas crypt depth was determined in the colon from the crypt base to the luminal surface. An average of 30 well-oriented villi or crypts per animal was analyzed, and morphometric analyses were performed using ImageJ Version 1.54p (National Institutes of Health, Bethesda, MD, USA).

### 2.10. Oil Red O Staining

Lipid accumulation in the liver was evaluated by Oil Red O (ORO) staining. Liver samples were embedded in Tissue-Tek^®^ (Thermo Fisher Scientific, Waltham, MA, USA), snap-frozen in liquid nitrogen, sectioned at 12 μm using a cryostat (Microm H560), and stained with Oil Red O. Three sections from different tissue regions were placed on each slide, with two slides prepared per animal. Ten images per animal were acquired using a Nikon Eclipse Ti-U microscope coupled to a Nikon DS-Ri1 digital camera (20× objective). Lipid-positive area was quantified using ImageJ (National Institutes of Health, Bethesda, MD, USA).

### 2.11. Picrosirius Red Staining

Hepatic collagen deposition was evaluated by Picrosirius Red staining. Paraffin sections (5 μm) were stained with Picrosirius Red, and 10 images per animal from three different sections were acquired using a 20× objective. Collagen deposition was quantified using ImageJ (National Institutes of Health, Bethesda, MD, USA) and expressed as the percentage of the total tissue area.

### 2.12. Western Blot

Protein expression in liver samples was evaluated by Western blot. Approximately 50–100 mg of liver tissue was homogenized in ice-cold RIPA buffer containing protease and phosphatase inhibitors using a Polytron PTA 20S homogenizer (Brinkmann Instruments). Homogenates were centrifuged at 12,000 rpm (15,294 rcf) for 20 min at 4 °C, and protein concentration was determined by the Bradford assay (Bio-Rad Laboratories, Hercules, CA, USA). Next, 30 μg of protein was separated by SDS-PAGE, transferred to PVDF membranes (Bio-Rad), and blocked with 5% non-fat milk or bovine serum albumin (BSA), depending on the primary antibody. Membranes were incubated overnight at 4 °C with primary antibodies against Gal-3, JNK, phospho-JNK, AKT2, and phospho-AKT2^ser474^ (Cell Signaling Technology, Danvers, MA, USA; Signalway Antibody, USA), followed by incubation with horseradish peroxidase-conjugated secondary antibodies. Immunoreactive bands were detected by enhanced chemiluminescence (ECL Plus, Amersham, ThermoFisher Scientific, Waltham, MA, USA), quantified by densitometry using Scion Image (Scion Corporation, Frederick, MD, USA), and normalized to Ponceau S staining.

### 2.13. Immunohistochemistry

Hepatic macrophage infiltration was assessed by F4/80 immunohistochemistry. Liver samples were fixed in 10% formaldehyde for 8 h, paraffin-embedded, and sectioned at 5 μm. After deparaffinization and rehydration, antigen retrieval was performed in 10 mM sodium citrate buffer (pH 6.0) containing 0.05% Tween 20 at 98 °C for 10 min. Endogenous peroxidase activity was blocked with 1% hydrogen peroxide, and nonspecific binding was blocked with goat serum containing bovine serum albumin (BSA). Sections were incubated overnight at 4 °C with a rabbit anti-F4/80 antibody (Bio-Rad AbD Serotec, Kidlington, UK; 1:200) diluted in PBS containing 0.3% Triton X-100, followed by incubation with a biotinylated secondary antibody and the Vectastain ABC Kit (Vector Laboratories, Burlingame, CA, USA). Immunoreactivity was visualized using 3,3′-diaminobenzidine (DAB) and counterstained with Harris hematoxylin. Negative controls were performed by omitting the primary antibody. Images were acquired using a Nikon Eclipse E600 microscope coupled to an Olympus DP-72 digital camera. At least five fields per section were analyzed in a blinded manner using Image-Pro Plus 4.5 (Media Cybernetics, Bethesda, MD, USA), and F4/80 expression was reported as the percentage of positively stained area relative to the total tissue area.

### 2.14. Total RNA Extraction and Real-Time Quantitative PCR

F4/80 mRNA expression in the liver was quantified by real-time quantitative PCR (RT-qPCR). Approximately 50 mg of snap-frozen liver tissue was homogenized in 1 mL of TRIzol^®^ reagent (Life Technologies, Carlsbad, CA, USA) for total RNA extraction. RNA concentration and purity were measured using a NanoDrop spectrophotometer (Thermo Scientific, Waltham, MA, USA) based on absorbance at 260 nm and the 260/280 nm ratio. Complementary DNA (cDNA) was synthesized using the High-Capacity cDNA Reverse Transcription Kit (Applied Biosystems, Waltham, MA, USA). RT-qPCR was performed on a Rotor-Gene Q system (Qiagen, Hilden, Germany) with Platinum^®^ SYBR^®^ Green qPCR SuperMix UDG (Invitrogen, Waltham, MA, USA). F4/80 expression was normalized to β-actin. The F4/80 primer sequences were 5′-CCTGGACGAATCCTGTGAAG-3′ (forward) and 5′-GGTGGGACCACAGAGAGTTG-3′ (reverse).

### 2.15. Use of Generative Artificial Intelligence

Figures 1 and 8 were initially designed by the authors using BioRender. Generative artificial intelligence was subsequently used to assist with the graphical refinement and modification of selected illustrative elements, based on specific instructions provided by the authors. The experimental design, scientific content, selection and interpretation of the results represented, and the final organization of the figures were determined by the authors. All AI-assisted modifications were reviewed and approved by the authors. Generative AI was not used to generate, modify, analyze, or interpret experimental data.

### 2.16. Statistical Analysis

Data were organized using Microsoft Excel (Microsoft Corporation, Redmond, WA, USA), and statistical analyses were performed using GraphPad Prism version 10.0 (GraphPad Software, San Diego, CA, USA). Data are presented as mean ± SEM. Comparisons among experimental groups were performed using two-way ANOVA followed by Tukey’s multiple comparisons test. Statistical significance was set at *p* < 0.05. Depending on the analysis, each experimental group consisted of 4–10 animals. Histological analyses were performed in a blinded manner. Liver morphology was quantified from five representative fields per animal for H&E and F4/80 immunohistochemistry, and from twenty fields acquired from three different sections per animal for Oil Red O and Picrosirius Red staining. Intestinal morphometry was performed by analyzing an average of 30 well-oriented villi or crypts per animal. Weekly food and water intake were measured per cage, normalized to the number of animals, and are presented as descriptive data without inferential statistical analysis.

## 3. Results

### 3.1. Impact of Western Diet and Gal-3 Inhibition on Body Weight and Body Composition

**Figure 2:**
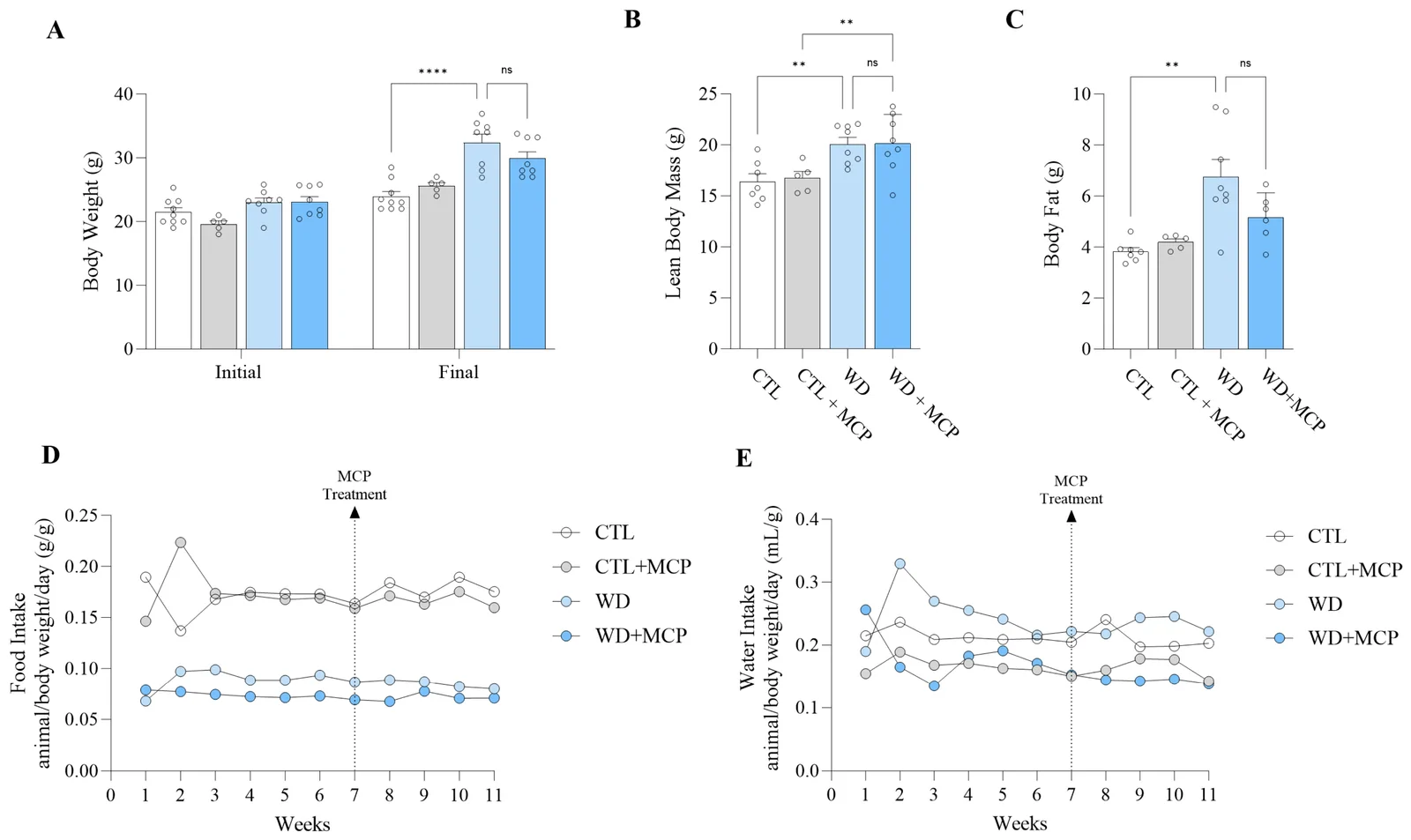
Effects of Western diet and Gal-3 inhibition on body weight, body composition, and weekly food and water intake in ApoE-deficient mice. (**A**) Baseline and endpoint body weight. (**B**) Lean body mass at the end of the experimental protocol. (**C**) Fat body mass at the end of the experimental protocol. (**D**) Weekly food intake normalized per mouse. (**E**) Weekly water intake normalized per mouse. The dotted line indicates the beginning of MCP treatment. For panels (**A**–**C**), data are presented as mean ± SEM, with each dot representing one mouse (n = 5–8 per group, depending on the analysis). Statistical significance was determined by two-way ANOVA followed by Tukey’s multiple comparisons test. For panels (**D**,**E**), food and water intake were measured weekly per cage and normalized to the number of mice housed in each cage. Because one cage was used per experimental group, these data are presented descriptively and were not subjected to inferential statistical analysis. ns, not significant; ** *p* < 0.01, **** *p* < 0.0001.

Western diet (WD) feeding significantly increased final body weight in both untreated and MCP-treated ApoE-deficient mice compared with their respective chow-fed controls (Figure 2A). Overall, WD feeding resulted in an approximately 35% increase in body weight relative to chow-fed mice. Body composition analysis revealed that lean mass was significantly greater in WD mice than in CTL mice and in WD + MCP mice than in CTL + MCP mice (Figure 2B). Likewise, fat mass was markedly increased in WD mice, showing an approximately 77% increase compared with CTL mice (Figure 2C), whereas no significant difference was observed between WD + MCP and CTL + MCP mice. Importantly, MCP treatment did not significantly affect body weight, lean mass, or fat mass in Western diet-fed mice, indicating that Gal-3 inhibition did not alter the effects of the Western diet on overall body composition.

Weekly food and water intake throughout the experimental protocol are presented in Figure 2D,E. Descriptive analysis revealed no apparent changes in food or water consumption during the study, including after the initiation of MCP treatment. Because intake was measured per cage and normalized to the number of animals per cage, these data are presented descriptively.

### 3.2. Effect of Gal-3 Inhibition on Glucose Homeostasis and Hepatic Insulin Signaling in Western Diet-Fed Mice

**Figure 3:**
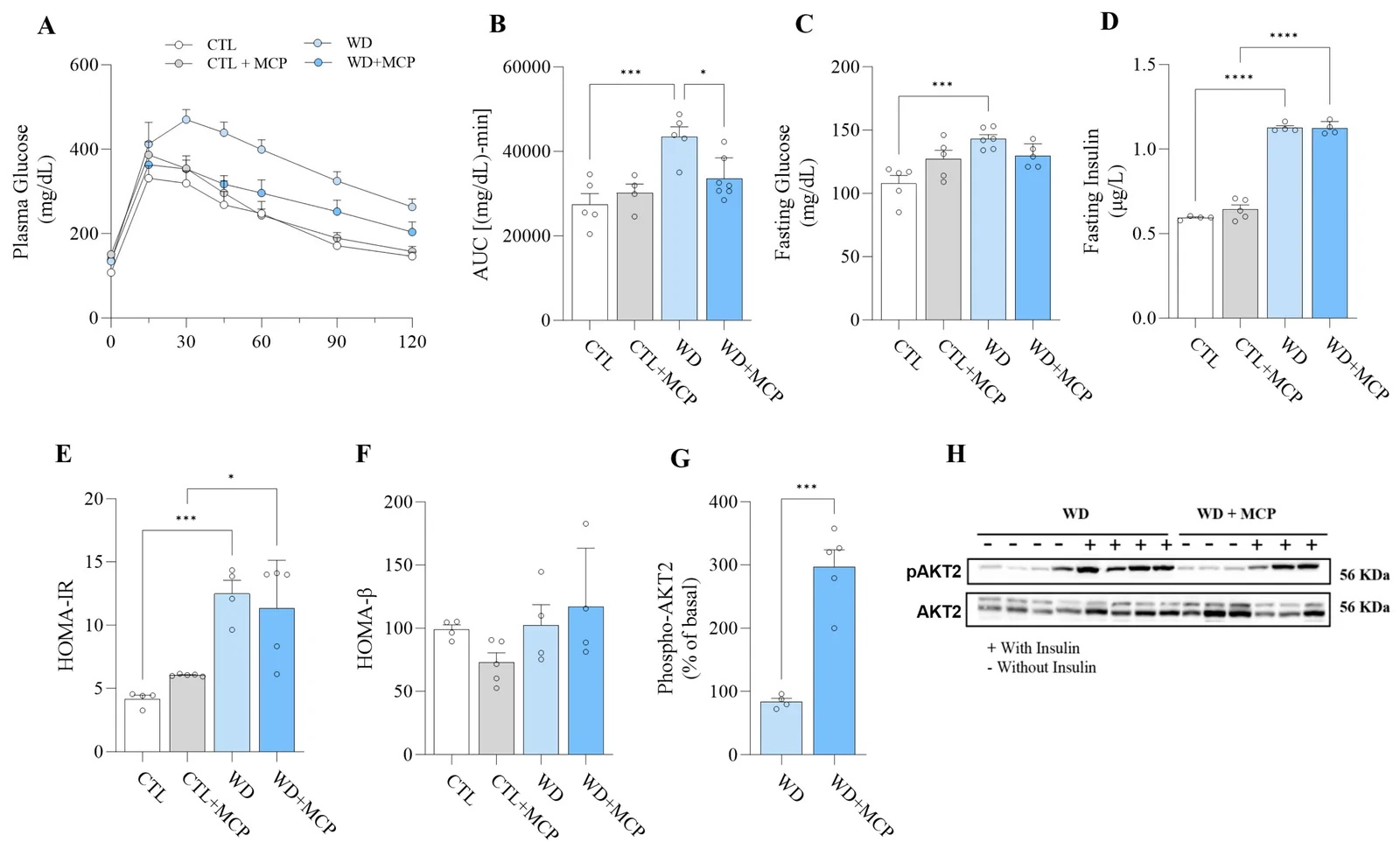
Effects of Western diet and Gal-3 inhibition on glucose homeostasis and hepatic insulin signaling in ApoE-deficient mice. (**A**) Intraperitoneal glucose tolerance test (ipGTT). Blood glucose concentrations were measured at the indicated time points following intraperitoneal glucose administration. (**B**) Area under the glucose tolerance curve (AUC). (**C**) Fasting blood glucose concentrations. (**D**) Fasting plasma insulin concentrations. (**E**) Homeostasis Model Assessment of Insulin Resistance (HOMA-IR). (**F**) Homeostasis Model Assessment of β-cell function (HOMA-β). (**G**) Quantification of insulin-stimulated AKT2 phosphorylation expressed as a percentage of basal levels. (**H**) Representative immunoblots of phospho-AKT2 (Ser474) and total AKT2 in liver samples collected before (−) and 30 s after (+) insulin stimulation. Data are presented as mean ± SEM (n = 3–7 animals per group). Statistical significance was determined by two-way ANOVA followed by Tukey’s multiple comparisons test. * *p* < 0.05, *** *p* < 0.001, **** *p* < 0.0001.

To evaluate the metabolic effects of MCP treatment, glucose tolerance, fasting glycemic parameters, and hepatic insulin signaling were assessed (Figure 3). WD-fed mice exhibited impaired glucose tolerance, as evidenced by higher blood glucose concentrations throughout the glucose tolerance test (Figure 3A) and a ~58% increase in the glucose area under the curve (AUC) compared with CTL mice (Figure 3B). MCP treatment significantly improved glucose tolerance, reducing the AUC by approximately 22% relative to untreated WD mice.

Fasting blood glucose was approximately 32% higher in WD mice than in CTL mice (Figure 3C). Similarly, fasting plasma insulin concentrations were increased by ~62% in WD mice compared with CTL mice and remained elevated in WD + MCP mice (~74% vs. CTL + MCP; Figure 3D). Consistent with these findings, HOMA-IR was increased by ~200% in WD mice relative to CTL mice and by ~86% in WD + MCP mice relative to CTL + MCP mice (Figure 3E), whereas HOMA-β did not differ among the experimental groups (Figure 3F).

To further assess hepatic insulin signaling, AKT2 phosphorylation was evaluated following acute insulin stimulation. MCP treatment markedly enhanced insulin-stimulated AKT2 phosphorylation, with WD + MCP mice exhibiting a ~254% increase compared with untreated WD mice (Figure 3G,H). These findings indicate that Gal-3 inhibition improves hepatic insulin signaling despite the persistence of elevated fasting insulin concentrations and HOMA-IR.

### 3.3. Effects of Western Diet and Gal-3 Inhibition on Hepatic Lipid Accumulation and Plasma Lipid Profile

**Figure 4:**
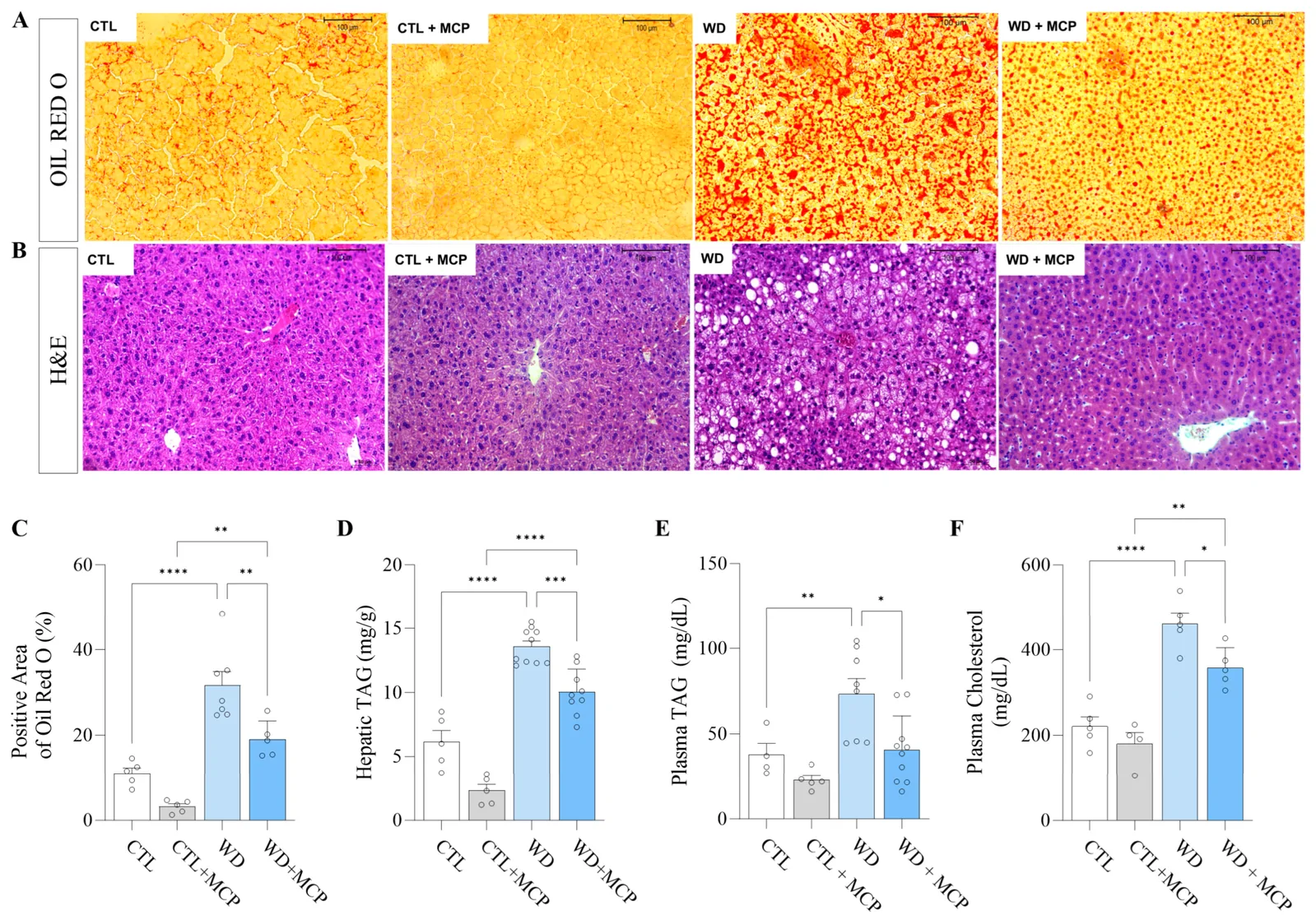
Effects of Western diet and Gal-3 inhibition on hepatic lipid accumulation and plasma lipid profile in ApoE-deficient mice. (**A**) Representative Oil Red O-stained liver sections showing neutral lipid accumulation. (**B**) Representative hematoxylin and eosin (H&E)-stained liver sections. (**C**) Quantification of Oil Red O-positive area. (**D**) Hepatic triglyceride content. (**E**) Plasma triglyceride concentrations. (**F**) Plasma total cholesterol concentrations. 20× objective; scale bars = 100 μm. Data are presented as mean ± SEM; n = 4–10 animals per group. Statistical significance was determined by two-way ANOVA followed by Tukey’s multiple comparisons test. * *p* < 0.05, ** *p* < 0.01, *** *p* < 0.001, **** *p* < 0.0001.

To determine whether Gal-3 inhibition also affects hepatic lipid accumulation and systemic lipid metabolism, hepatic steatosis and circulating lipid levels were evaluated (Figure 4). Representative Oil Red O staining revealed marked hepatic lipid accumulation in WD mice, which was visibly attenuated following MCP treatment (Figure 4A). Quantitative analysis demonstrated that the Oil Red O-positive area was 2.87-fold higher in WD mice than in CTL mice and 5.65-fold higher in WD + MCP mice than in CTL + MCP mice (Figure 4C). Importantly, MCP treatment reduced hepatic lipid accumulation by approximately 40% in Western diet-fed mice compared with untreated WD animals.

Consistent with these findings, hepatic triglyceride content was 2.21-fold higher in WD mice than in CTL mice and 4.32-fold higher in WD + MCP mice than in the corresponding CTL + MCP group (Figure 4D). Nevertheless, MCP treatment reduced hepatic triglyceride content by approximately 26% relative to untreated WD mice. Representative hematoxylin and eosin (H&E) staining further confirmed the marked hepatic steatosis induced by the Western diet and its attenuation following MCP treatment (Figure 4B).

Plasma triglyceride concentrations were approximately 94% higher in WD mice than in CTL mice, whereas no significant difference was observed between WD + MCP and CTL + MCP mice. Moreover, MCP treatment reduced plasma triglyceride concentrations by approximately 44% compared with untreated WD mice (Figure 4E). Similarly, plasma total cholesterol concentrations were increased by approximately 108% in WD mice relative to CTL mice and by approximately 98% in WD + MCP mice relative to CTL + MCP mice. However, MCP treatment reduced plasma cholesterol concentrations by approximately 22% compared with untreated WD mice (Figure 4F). Collectively, these findings demonstrate that MCP attenuates hepatic lipid accumulation and partially improves the dyslipidemia induced by Western diet feeding.

### 3.4. Effects of Western Diet and Gal-3 Inhibition on Hepatic Inflammation, Fibrosis, and Liver Injury

**Figure 5:**
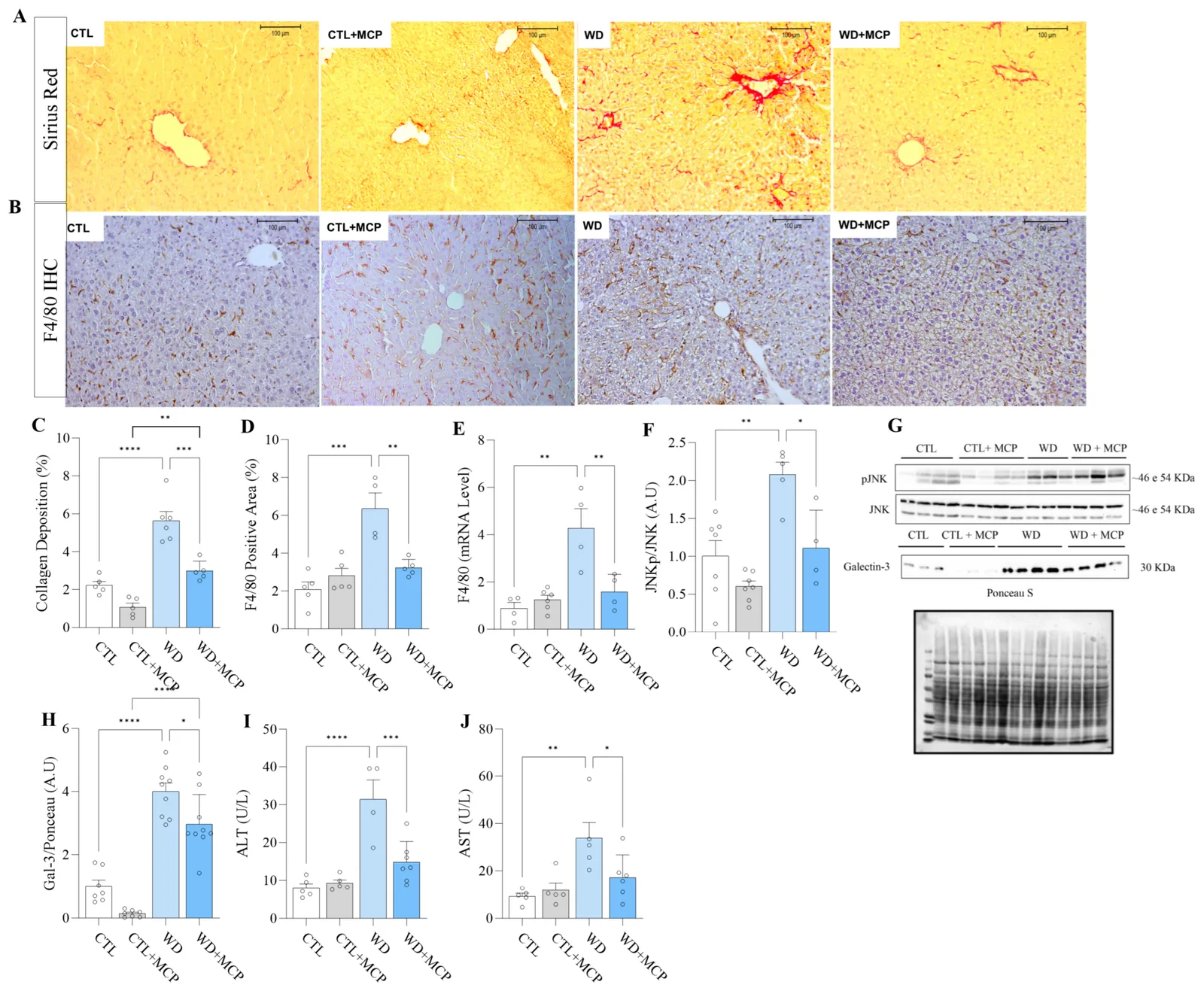
Effects of Western diet and Gal-3 inhibition on hepatic inflammation, fibrosis, and liver injury in ApoE-deficient mice. Representative Sirius Red staining showing hepatic collagen deposition (**A**) and representative F4/80 immunohistochemical staining showing hepatic macrophage infiltration (**B**). Quantification of hepatic collagen deposition (**C**), F4/80-positive area (**D**), hepatic F4/80 mRNA expression (**E**), hepatic phospho-JNK/JNK protein expression (**F**), representative immunoblots of phospho-JNK (pJNK), total JNK, Gal-3, and Ponceau S used as the loading control (**G**), hepatic Gal-3 protein expression normalized to Ponceau S (**H**), plasma alanine aminotransferase (ALT) activity (**I**), and plasma aspartate aminotransferase (AST) activity (**J**). 20× objective, scale bars = 100 μm. Data are presented as mean ± SEM (n = 4–9 animals per group). Statistical analyses were performed using two-way ANOVA followed by Tukey’s multiple comparisons test. * *p* < 0.05, ** *p* < 0.01, *** *p* < 0.001, **** *p* < 0.0001.

To investigate the effects of Gal-3 inhibition on hepatic inflammation, fibrosis, and liver injury, collagen deposition, macrophage infiltration, inflammatory signaling, hepatic Gal-3 expression, and circulating markers of liver injury were evaluated (Figure 5). Representative Sirius Red staining revealed marked collagen accumulation in the livers of WD-fed mice, which was attenuated following MCP treatment (Figure 5A). Consistent with these observations, collagen deposition increased by approximately 153% in WD mice compared with CTL mice and by approximately 178% in WD + MCP mice compared with CTL + MCP mice. Importantly, MCP treatment reduced collagen deposition by approximately 47% relative to untreated WD mice (Figure 5C).

Representative F4/80 immunohistochemistry demonstrated increased hepatic macrophage infiltration in WD mice, which was markedly attenuated following MCP treatment (Figure 5B). Accordingly, the F4/80-positive area increased by approximately 204% in WD mice compared with CTL mice and was reduced by approximately 49% in WD + MCP mice relative to untreated WD mice (Figure 5D). Likewise, hepatic F4/80 mRNA expression was approximately 4.8-fold higher in WD mice than in CTL mice and decreased by approximately 62% following MCP treatment (Figure 5E).

To further characterize hepatic inflammatory signaling, the phospho-JNK/JNK ratio was evaluated. Western diet feeding increased JNK phosphorylation by approximately 106% compared with CTL mice, whereas MCP treatment reduced the phospho-JNK/JNK ratio by approximately 46% relative to untreated WD mice (Figure 5F,G). Consistent with these findings, hepatic Gal-3 protein expression was approximately fourfold higher in WD mice than in CTL mice. Importantly, MCP treatment significantly reduced hepatic Gal-3 protein expression by approximately 25% compared with untreated WD mice (Figure 5H).

Consistent with the attenuation of hepatic inflammation and fibrosis, plasma ALT activity was increased by approximately 294% in WD mice compared with CTL mice and reduced by approximately 53% following MCP treatment (Figure 5I). Likewise, plasma AST activity was elevated by approximately 266% in WD mice relative to CTL mice and decreased by approximately 49% in WD + MCP mice compared with untreated WD mice (Figure 5J). Collectively, these findings demonstrate that Gal-3 inhibition attenuates Western diet-induced hepatic inflammation, fibrosis, inflammatory signaling, and liver injury.

### 3.5. Effects of Western Diet and Gal-3 Inhibition on Oral Lipid Tolerance and Fecal Triglyceride Excretion

**Figure 6:**
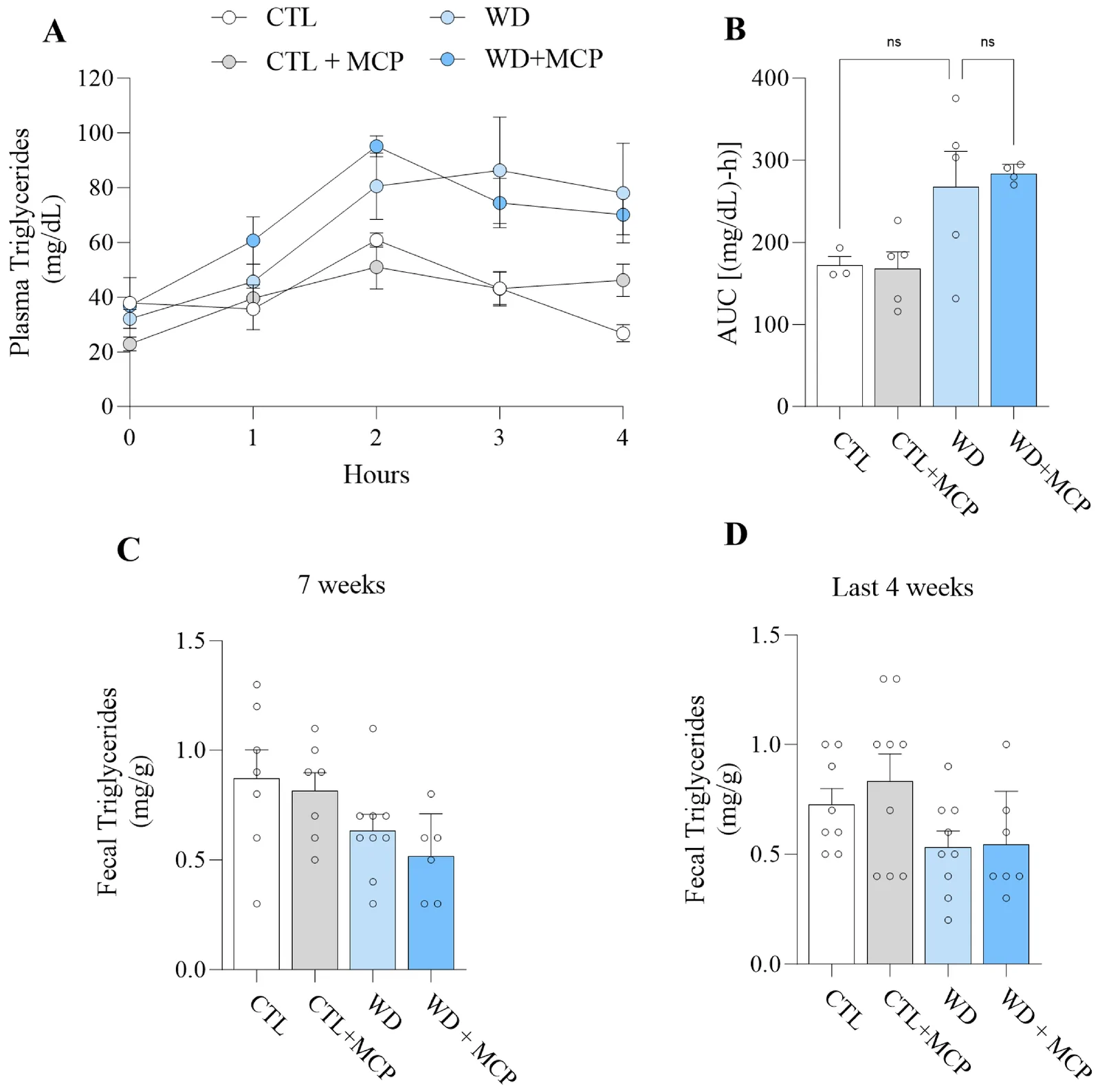
Effects of Western diet and Gal-3 inhibition on oral lipid tolerance and fecal triglyceride excretion in ApoE-deficient mice. (**A**) Plasma triglyceride concentrations measured during the oral lipid tolerance test (OLTT) following administration of 400 μL of lard by oral gavage, with blood samples collected at 0, 1, 2, 3, and 4 h. (**B**) Area under the curve (AUC) calculated from the OLTT. (**C**) Fecal triglyceride content after the initial 7 weeks of dietary intervention, before MCP treatment. (**D**) Fecal triglyceride content during the last 4 weeks of the experimental protocol, corresponding to the MCP treatment period. Data are presented as mean ± SEM, with each dot representing one mouse (n = 3–9 per group, depending on the analysis). Statistical significance was determined by two-way ANOVA followed by Tukey’s multiple comparisons test. ns, not significant.

To investigate whether Gal-3 inhibition influences lipid handling, postprandial triglyceride clearance was evaluated using an oral lipid tolerance test (OLTT), and fecal triglyceride content was measured before and during MCP treatment (Figure 6). Plasma triglyceride concentrations during the OLTT (Figure 6A) and the corresponding area under the curve (Figure 6B) did not differ significantly among the experimental groups. Likewise, fecal triglyceride content measured after the initial seven weeks of dietary intervention (Figure 6C) and during the final four weeks of the experimental protocol (Figure 6D) was not significantly different among the groups. Collectively, these findings indicate that MCP treatment did not significantly affect postprandial lipid clearance or fecal triglyceride excretion under the experimental conditions evaluated.

### 3.6. Effects of Western Diet and Gal-3 Inhibition on Intestinal Morphology

**Figure 7:**
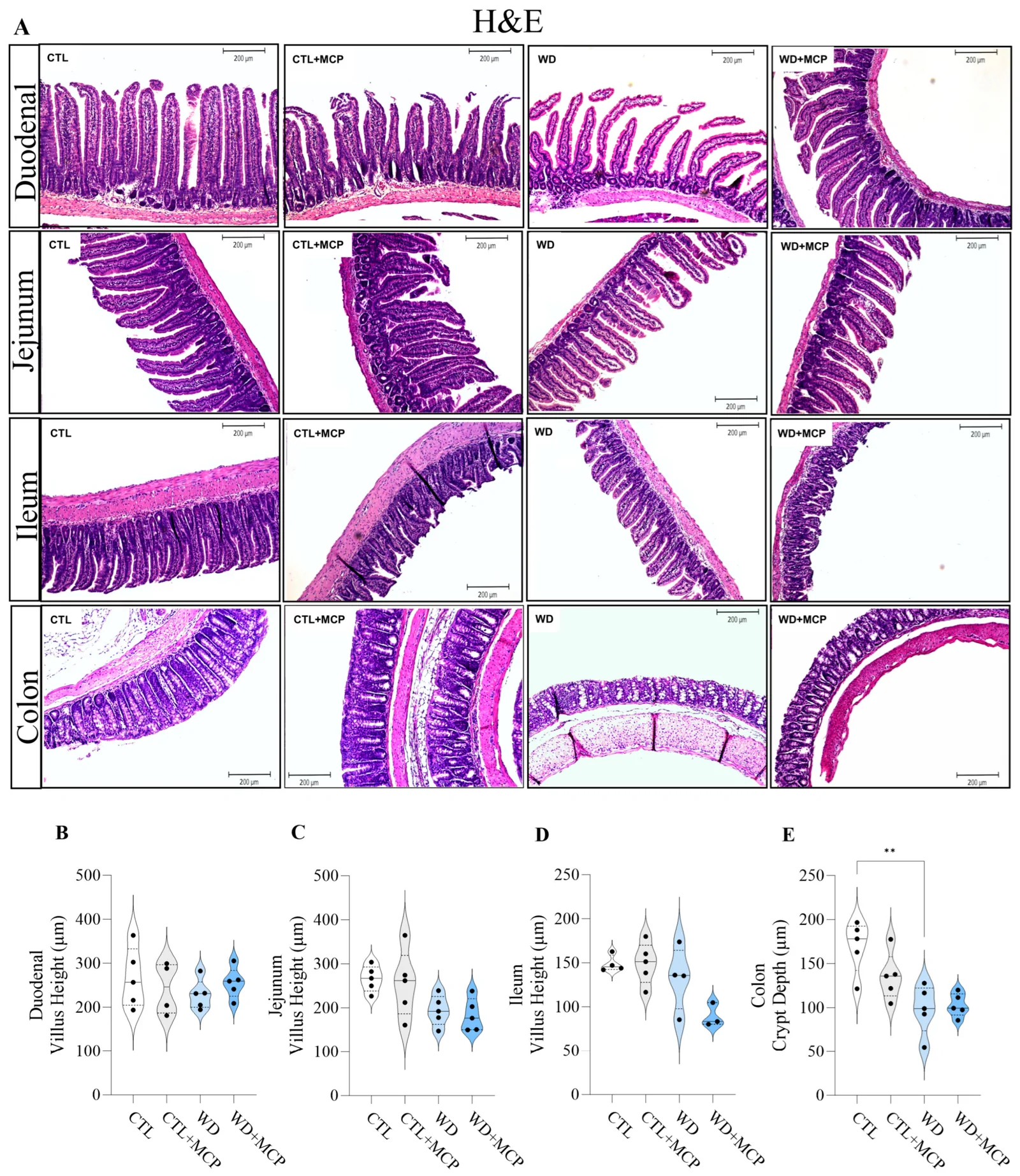
Effects of Western diet and Gal-3 inhibition on intestinal morphology in ApoE-deficient mice. (**A**) Representative hematoxylin and eosin (H&E)-stained sections of the duodenum, jejunum, ileum, and colon (10× objective; scale bar = 200 μm). (**B**) Duodenal villus height. (**C**) Jejunal villus height. (**D**) Ileal villus height. (**E**) Colonic crypt depth. Data are presented as mean ± SEM, with each dot representing one mouse (n = 5–8 per group, depending on the analysis). Statistical significance was determined by two-way ANOVA followed by Tukey’s multiple comparisons test. ** *p* < 0.01.

To determine whether oral MCP administration affected intestinal morphology, histological sections of the duodenum, jejunum, ileum, and colon were stained with hematoxylin and eosin (H&E) (Figure 7A). Villus height was measured in the duodenum, jejunum, and ileum, whereas crypt depth was quantified in the colon. No significant differences in villus height were observed among the experimental groups in any region of the small intestine (Figure 7B–D). In contrast, colonic crypt depth was approximately 42% lower in WD mice than in CTL mice (Figure 7E). However, no significant differences were detected between WD and WD + MCP mice, indicating that MCP treatment did not significantly alter intestinal morphology under the experimental conditions evaluated.

## 4. Discussion

MASH is a multifactorial disease characterized by profound metabolic dysfunction, chronic inflammation, progressive fibrosis, and ultimately liver injury. Despite advances in our understanding of its pathophysiology, effective pharmacological therapies remain limited, underscoring the need to identify novel therapeutic targets capable of simultaneously modulating the metabolic and inflammatory pathways that contribute to disease progression. In the present study, we investigated whether pharmacological inhibition of Gal-3 with modified citrus pectin (MCP) could attenuate established MASH in ApoE-deficient mice. Importantly, MCP treatment was initiated after seven weeks of Western diet feeding, when metabolic dysfunction and hepatic steatosis were already established, thereby allowing us to assess the therapeutic, rather than preventive, effects of Gal-3 inhibition. Overall, our findings demonstrate that MCP treatment significantly improved glucose homeostasis, hepatic insulin signaling, steatosis, inflammation, fibrosis, and liver injury induced by Western diet feeding, supporting Gal-3 as a promising therapeutic target for MASH.

Chronic caloric excess is a major driver of MASH development, promoting obesity, insulin resistance, dyslipidemia, and ectopic lipid accumulation in the liver. Consistent with this concept, ApoE-deficient mice fed a Western diet supplemented with fructose developed the expected metabolic phenotype, characterized by increased body weight, adiposity, and lean mass. Although Western diet-fed mice consumed less food by weight than control animals, they exhibited greater body weight gain and fat accumulation, likely reflecting the higher caloric density of the Western diet rather than differences in food intake. Water consumption remained unchanged throughout the study and was unaffected by MCP treatment, indicating that the metabolic improvements observed with MCP were not attributable to differences in fluid intake or, consequently, MCP exposure.

Accumulating clinical evidence indicates that Gal-3 is closely associated with obesity and metabolic dysfunction. Circulating Gal-3 concentrations positively correlate with body mass index, insulin resistance, type 2 diabetes, dyslipidemia, and multiple cardiometabolic risk factors in humans [35,36,37]. Similarly, hepatic Gal-3 expression is markedly increased in patients with MASH and in experimental models of metabolic liver disease, where it has been implicated in inflammatory cell recruitment and fibrogenesis [22]. Consistent with these observations, Western diet feeding markedly increased hepatic Gal-3 protein abundance in the present study. Interestingly, despite substantially improving hepatic pathology and glucose metabolism, MCP treatment did not alter body weight or body composition. Similar findings have been reported in previous studies describing minimal effects of MCP on adiposity [38]. These observations suggest that the beneficial metabolic and hepatoprotective effects of MCP occur independently of changes in body weight, reinforcing the concept that targeting inflammatory pathways may ameliorate MASH even in the absence of weight loss.

Insulin resistance is a central feature of MASH and plays a pivotal role in the development of hepatic steatosis by promoting hyperglycemia, hyperinsulinemia, increased adipose tissue lipolysis, and excessive delivery of non-esterified fatty acids (NEFAs) to the liver [39,40,41]. Consistent with this concept, Western diet-fed ApoE-deficient mice developed marked glucose intolerance accompanied by elevated fasting glucose, fasting insulin, and HOMA-IR, confirming the presence of systemic metabolic dysfunction. Remarkably, MCP treatment significantly improved glucose tolerance and enhanced insulin-stimulated hepatic AKT2 phosphorylation, indicating improved hepatic insulin signaling. Although fasting insulin concentrations and HOMA-IR were only modestly affected, these findings are not necessarily contradictory. HOMA-IR primarily reflects systemic insulin resistance under basal conditions, whereas insulin-stimulated AKT2 phosphorylation provides a more direct assessment of hepatic insulin signaling. Thus, the improvement in hepatic insulin responsiveness following MCP treatment may precede detectable changes in systemic indices of insulin resistance. Importantly, the metabolic benefits of MCP may also involve effects on adipose tissue. MCP-mediated attenuation of adipose tissue inflammation could reduce adipocyte lipolysis and, consequently, decrease the flux of NEFAs from adipose tissue to the liver [42,43]. Reduced hepatic NEFA delivery may limit hepatic lipid accumulation and thereby contribute to the improvement in hepatic insulin signaling. This potential adipose tissue–liver axis provides a possible explanation for the improvement in glucose homeostasis and hepatic steatosis despite the absence of significant changes in body weight, lean mass, or fat mass following MCP treatment. However, this mechanism remains speculative and warrants further investigation.

Accumulating evidence identifies Gal-3 as an important regulator of glucose homeostasis and insulin action. In addition to promoting inflammatory responses in metabolic tissues, Gal-3 has been implicated in pancreatic β-cell dysfunction, macrophage activation, and impaired insulin signaling in the context of obesity and type 2 diabetes [23]. Consistent with this concept, genetic reduction or deletion of Gal-3 has been shown to improve glucose tolerance and systemic insulin sensitivity in obese mice, including enhanced hepatic insulin sensitivity and increased insulin-stimulated AKT phosphorylation [44]. Moreover, Li et al. [44] demonstrated that Gal-3 can directly interact with the insulin receptor and impair insulin-induced receptor tyrosine phosphorylation, with consequent attenuation of downstream IRS1, PDK1, and AKT signaling. These findings provide a plausible molecular mechanism by which increased Gal-3 may contribute directly to insulin resistance in hepatocytes and other insulin-sensitive tissues. Therefore, the reduction in hepatic Gal-3 expression following MCP treatment may have contributed to the restoration of hepatic insulin signaling and the improvement in glucose tolerance observed in the present study. However, given that the present study did not directly assess Gal-3–insulin receptor interaction or functional Gal-3 activity, this interpretation remains mechanistic and should be considered a potential rather than definitively established explanation for the observed effects. Although the precise molecular mechanisms underlying these effects remain to be established, our findings further support the concept that Gal-3 may represent an important molecular link between chronic inflammation and metabolic dysfunction.

Hepatic steatosis is a histological hallmark of MASH and reflects a chronic imbalance between hepatic lipid acquisition and disposal. Under conditions of nutrient excess and insulin resistance, triglycerides accumulate within hepatocytes through multiple complementary mechanisms, including increased NEFA influx from adipose tissue, enhanced de novo lipogenesis, impaired fatty acid oxidation, and reduced very-low-density lipoprotein (VLDL) secretion [42,43,45]. Accordingly, Western diet feeding markedly increased hepatic lipid accumulation and circulating cholesterol concentrations in ApoE-deficient mice, confirming the development of pronounced metabolic dysfunction. Importantly, MCP treatment substantially reduced both hepatic neutral lipid deposition and triglyceride content despite continued exposure to the Western diet. These improvements occurred independently of changes in body weight or adiposity, indicating that the reduction in steatosis was not secondary to weight loss but instead reflected improved hepatic metabolic homeostasis. These findings are consistent with previous evidence implicating Gal-3 in the regulation of hepatic lipid accumulation. Iacobini et al. [46] reported that Gal-3-deficient mice developed markedly less hepatic steatosis despite exhibiting increases in circulating lipids comparable to those observed in wild-type animals. Gal-3 deficiency was also associated with reduced expression of several genes involved in hepatic lipid synthesis and oxidation, as well as lower hepatic CD36 expression [46]. These findings support the possibility that modulation of the Gal-3 pathway may influence hepatic lipid handling independently of changes in systemic lipid availability and are consistent with the reduction in hepatic steatosis observed following MCP treatment in the present study. Recent evidence further supports a potential direct role of Gal-3 in the regulation of hepatic lipid accumulation. Using a human multilineage 3D hepatic spheroid model composed of HepG2 and hepatic stellate cells, Sedda et al. [47] demonstrated that LGALS3 silencing markedly reduced intracellular neutral lipid accumulation and was accompanied by changes in genes involved in triglyceride synthesis and fatty acid metabolism, including DGAT1, CPT1A, and PPARA, without major alterations in genes related to lipid uptake and transport [47]. These findings provide independent evidence that modulation of the Gal-3 pathway can influence hepatic lipid handling and support the possibility that the reduction in hepatic steatosis observed following MCP treatment may, at least in part, involve changes in Gal-3-associated metabolic pathways. However, the precise metabolic mechanisms responsible for the reduction in hepatic lipid accumulation in our in vivo model remain to be established.

The concomitant improvement in steatosis and insulin signaling is biologically plausible, as these processes are closely interconnected. Excessive intracellular lipid accumulation promotes the generation of bioactive lipid intermediates, particularly diacylglycerols and ceramides, which can impair insulin signaling through activation of stress-responsive kinases and disruption of downstream insulin signaling pathways [48,49,50]. Conversely, restoration of hepatic insulin signaling can suppress hepatic glucose production and improve whole-body metabolic homeostasis, while indirectly limiting adipose tissue lipolysis and the consequent delivery of NEFAs to the liver. Thus, the parallel improvements in glucose tolerance, hepatic insulin signaling, and lipid accumulation observed following MCP treatment likely reflect a coordinated restoration of metabolic homeostasis rather than an isolated effect on hepatic lipid storage. Although the present study was not designed to determine the specific metabolic pathways responsible for the reduction in steatosis, future studies examining hepatic fatty acid uptake, de novo lipogenesis, mitochondrial β-oxidation, and VLDL secretion will be important for defining the mechanisms underlying the metabolic benefits of Gal-3 inhibition.

Persistent hepatic inflammation is a major determinant of MASH progression and represents a critical transition from simple steatosis to steatohepatitis and fibrosis [51]. Sustained lipid overload promotes hepatocellular injury and the release of damage-associated molecular patterns (DAMPs), triggering the activation of resident Kupffer cells and the recruitment of circulating monocyte-derived macrophages. These inflammatory cells amplify the hepatic inflammatory response through the production of cytokines and chemokines, further exacerbating hepatocellular dysfunction and promoting hepatic stellate cell activation. Sustained stellate cell activation ultimately leads to excessive extracellular matrix deposition and progressive fibrosis [51].

Gal-3 has emerged as an important regulator of this metabolic-inflammatory network. Beyond serving as a marker of macrophage activation, Gal-3 actively modulates macrophage polarization, inflammatory signaling, cell–cell communication, and hepatic stellate cell activation, thereby contributing to tissue remodeling and fibrogenesis [22,52]. Experimental studies have further shown that Gal-3 promotes transforming growth factor-β (TGF-β)-dependent profibrotic signaling, enhances collagen synthesis, and facilitates extracellular matrix accumulation during chronic liver injury. Consistent with these mechanisms, increased hepatic Gal-3 expression has been associated with progressive MASH and advanced liver fibrosis, supporting a potential role for Gal-3 in linking persistent hepatic inflammation to fibrogenesis.

Consistent with these mechanisms, Western diet feeding markedly increased hepatic macrophage accumulation, inflammatory signaling, collagen deposition, and Gal-3 expression in ApoE-deficient mice. Importantly, pharmacological inhibition of Gal-3 with MCP significantly attenuated each of these pathological features. The reduction in F4/80-positive macrophages, together with the marked decrease in hepatic F4/80 mRNA expression, indicates that MCP effectively limited macrophage accumulation in the liver during disease progression. Because activated macrophages are major sources of pro-inflammatory and profibrotic mediators, their reduction may have contributed to the attenuation of hepatic stellate cell activation and collagen deposition observed in the present study.

Concomitant with the reduction in macrophage accumulation, MCP treatment markedly decreased JNK phosphorylation. Activation of the JNK pathway is a well-established response to lipid overload and inflammatory stress and contributes to both hepatic insulin resistance and chronic inflammation by impairing insulin signaling and promoting inflammatory gene expression [43,48]. Thus, attenuation of JNK activation may represent an important mechanistic link between Gal-3 inhibition, improved hepatic insulin signaling, and reduced hepatic inflammation. Rather than occurring independently, these processes likely reinforce one another, disrupting the self-perpetuating cycle whereby lipid accumulation promotes inflammation, inflammation exacerbates insulin resistance, and insulin resistance further accelerates hepatic lipid accumulation.

The marked reduction in collagen deposition following MCP treatment further supports a beneficial effect of Gal-3 inhibition on the fibrogenic response during MASH progression. Hepatic fibrosis is the strongest histological predictor of liver-related morbidity and mortality in patients with MASH, underscoring the clinical relevance of interventions capable of limiting extracellular matrix accumulation. Our findings are consistent with previous pharmacological studies showing that targeting Gal-3 can attenuate hepatic fibrosis [53]. Traber and Zomer [53] reported that treatment with the Gal-3-binding compounds GR-MD-02 and GM-CT-01 markedly reduced collagen deposition and improved histological features of NASH in mice, including when treatment was initiated after fibrosis had been established. Notably, GR-MD-02 treatment was also associated with reduced α-smooth muscle actin (α-SMA) expression, supporting an effect on the fibrogenic response. Although the present study was not designed to directly assess hepatic stellate cell activation, the concomitant reduction in macrophage accumulation, inflammatory signaling, Gal-3 expression, and collagen deposition suggests that MCP may attenuate the cellular and molecular processes underlying fibrogenesis. Future studies assessing established markers of stellate cell activation and fibrogenic signaling, including α-smooth muscle actin (α-SMA), collagen type I, transforming growth factor-β (TGF-β), and tissue inhibitors of metalloproteinases (TIMPs), will be important to further define the molecular mechanisms through which Gal-3-associated pathways may limit hepatic fibrosis.

The histological improvements observed following MCP treatment were accompanied by significant reductions in circulating ALT and AST activities, indicating reduced hepatocellular injury. Because these enzymes are commonly used as biochemical indicators of hepatocellular damage, their reduction is consistent with the overall attenuation of hepatic inflammation and fibrosis and further supports the hepatoprotective effects of Gal-3 inhibition. Taken together, these findings support a model in which Gal-3 acts as an important molecular link connecting metabolic dysfunction, chronic inflammation, macrophage activation, fibrogenesis, and hepatocellular injury. Accordingly, pharmacological inhibition of Gal-3 may represent a promising therapeutic strategy for MASH by simultaneously targeting multiple interconnected pathological processes that contribute to disease progression.

Because MCP is administered orally and consists of a soluble dietary fiber, some of its metabolic effects could potentially result from alterations in intestinal nutrient handling. Soluble fibers can increase luminal viscosity, delay gastric emptying, and reduce the intestinal absorption of glucose and lipids. Accordingly, increased fecal lipid excretion and improved postprandial lipid handling have been proposed as potential mechanisms underlying the metabolic benefits of pectin supplementation. However, the effects of purified MCP on intestinal lipid absorption remain poorly characterized, and previous studies have reported inconsistent findings regarding its capacity to alter lipid metabolism or nutrient absorption [54].

In the present study, MCP treatment did not alter plasma triglyceride responses during the oral lipid tolerance test or fecal triglyceride excretion, suggesting that intestinal lipid absorption was not substantially affected under our experimental conditions. These findings are particularly relevant because they indicate that the marked reductions in hepatic steatosis and improvements in glucose homeostasis are unlikely to be explained by reduced dietary lipid absorption. Rather, they support the interpretation that the metabolic benefits of MCP are primarily associated with improvements in hepatic metabolic and inflammatory pathways, rather than with reduced intestinal lipid availability.

Histological evaluation further supported this interpretation. MCP treatment did not significantly alter villus height throughout the small intestine or crypt depth in the colon. Although the effects of obesogenic diets on intestinal morphology remain controversial, with previous studies reporting both reductions [55,56] and increases [57] in villus height and crypt dimensions, our findings indicate that overall intestinal architecture was largely preserved throughout the experimental protocol. Interestingly, Western diet feeding selectively reduced colonic crypt depth, partially recapitulating the findings reported by Xie et al. (2020) [55] and suggesting an alteration in colonic epithelial homeostasis in response to chronic dietary challenge. Nevertheless, this morphological change was not reversed by MCP treatment, further supporting the interpretation that the beneficial hepatic effects of MCP are unlikely to be primarily mediated by structural remodeling of the intestine.

## 5. Conclusions

This study has several limitations that should be acknowledged. First, although MCP is a well-characterized Gal-3-targeting polysaccharide and MCP treatment was associated with reduced hepatic Gal-3 protein expression and improved metabolic and hepatic outcomes, the present study did not directly assess MCP-Gal-3 target engagement, Gal-3 functional activity, or establish a causal relationship between Gal-3 inhibition and the observed effects. In addition, no selective Gal-3 comparator, genetic silencing, or Lgals3-deficient model was included, and the possibility that the reduction in hepatic Gal-3 expression was partly related to decreased macrophage abundance cannot be excluded. Second, the intestinal analyses were exploratory and limited to oral lipid tolerance, fecal triglyceride excretion, and gross intestinal morphology. Although these preliminary findings did not provide statistically significant evidence of effects on these parameters, they do not exclude potential contributions of other gut–liver axis mechanisms, including alterations in the microbiota, bile acid metabolism, intestinal permeability, or enterohepatic signaling. Third, food and water intake were assessed at the cage level using a single cage per experimental group, and therefore, these measurements could not be subjected to inferential statistical analysis or used to definitively exclude differences in caloric or MCP exposure between groups. Fourth, although most experiments included up to 10 animals per group, tissue availability prevented all molecular and biochemical analyses from being performed in every animal, resulting in smaller sample sizes for some specific endpoints and consequently limited statistical power. Finally, hepatic stellate cell activation was not directly assessed, and the fibrosis-related findings were based primarily on collagen deposition. Future studies incorporating direct assessment of stellate cell activation and fibrogenic signaling, as well as more comprehensive analyses of Gal-3 target engagement and the gut–liver axis, will be important to further define the mechanisms underlying the therapeutic effects of MCP in MASH.

Collectively, these findings provide further insight into the therapeutic effects of MCP and indicate that major alterations in the specific intestinal parameters evaluated are unlikely to account for its metabolic benefits. Rather, our findings support a model in which MCP is associated with improvements in hepatic metabolic signaling, inflammation, and fibrosis, with modulation of Gal-3-associated pathways representing a potential mechanism contributing to these effects.

**Figure 8:**
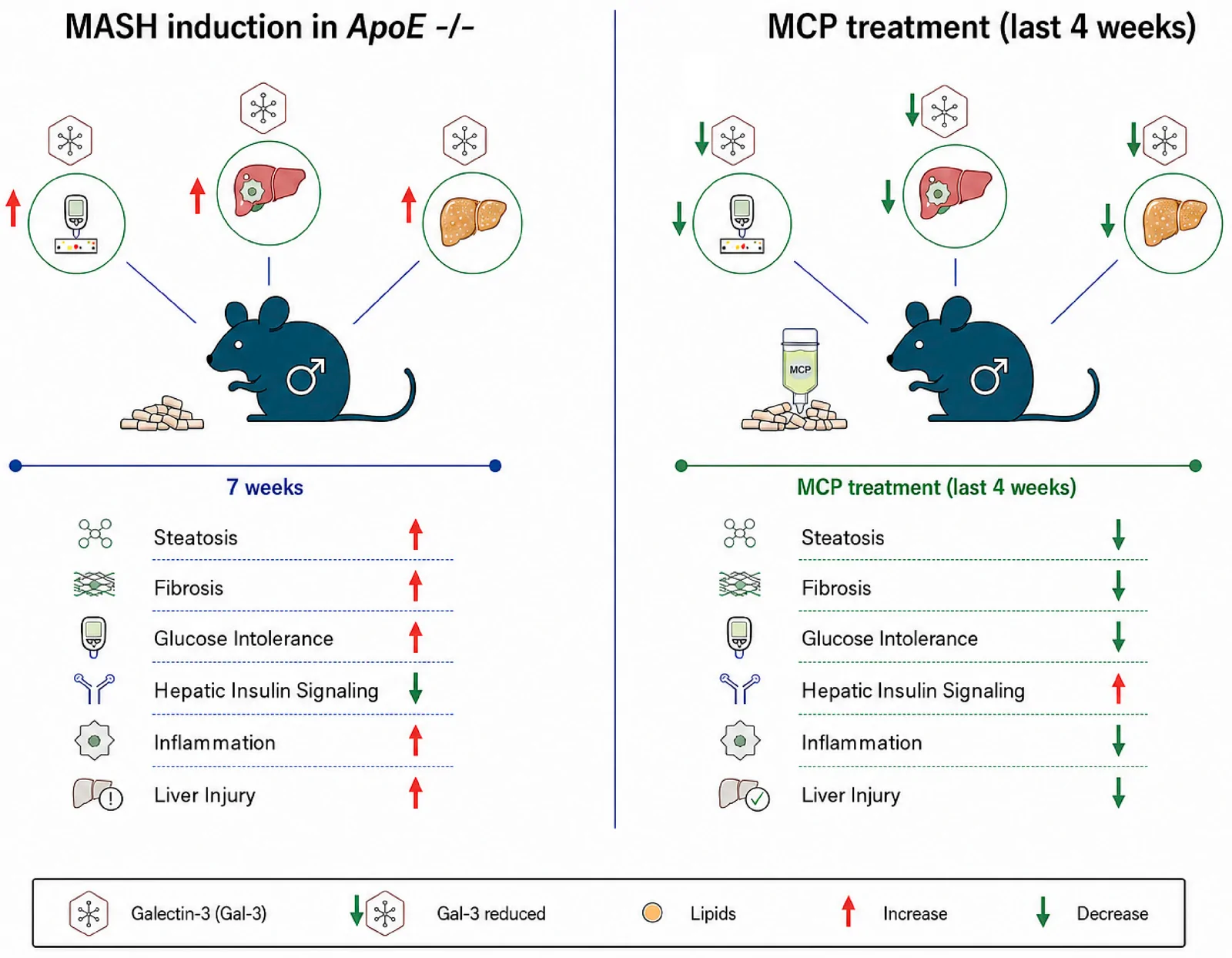
Graphical summary of the effects of MCP treatment and reduced Galectin-3 levels in ApoE −/− mice with MASH. Seven weeks of Western diet feeding induced hepatic steatosis, fibrosis, glucose intolerance, impaired hepatic insulin signaling, inflammation, and liver injury (left panel). MCP supplementation (1% in drinking water) during the last 4 weeks reduced Galectin-3 levels and attenuated these pathological alterations, decreasing hepatic lipid accumulation, fibrosis, glucose intolerance, inflammation, and liver injury while improving hepatic insulin signaling (right panel). Reduced Galectin-3 levels are represented by a downward arrow adjacent to the Gal-3 symbol. Red upward and green downward arrows indicate increased and decreased parameters, respectively.

Overall, the present study demonstrates that MCP attenuates several hallmark features of established MASH, including impaired glucose homeostasis, altered hepatic insulin signaling, steatosis, inflammation, fibrosis, and liver injury, as summarized in Figure 8. Importantly, these beneficial effects occurred without significant changes in body weight or adiposity, and no statistically significant evidence of an effect on the specific intestinal parameters evaluated, including intestinal morphology, oral lipid tolerance, or fecal triglyceride excretion, was detected under the conditions studied. These findings suggest that the metabolic and hepatic benefits of MCP are not readily explained by major alterations in the intestinal lipid-handling parameters assessed in this study. Although further studies are needed to establish MCP-Gal-3 target engagement, determine the precise molecular and cellular mechanisms involved, and assess the contribution of other potential gut–liver pathways, our findings provide preclinical evidence that MCP attenuates established MASH and support further investigation of Gal-3-associated signaling as a potential therapeutic target in metabolic liver disease.

## Figures and Tables

**Table 1:** Macronutrient composition is expressed as grams per 100 g of diet, and energy contribution is expressed as the percentage of total calories supplied by each macronutrient. Nutritional and energy values for the standard diet were obtained from published data for Nuvilab^®^ CR-1. Values for the Western diet were obtained or calculated from the D12079B formulation provided by Research Diets. ND: not declared as a single value; vitamins were specified individually by the manufacturer.

Nutrients (g/100 g Diet)	Standard Diet (Nuvilab^®^ CR-1)	Western Diet (D12079B)
Protein	22.50	19.77
Carbohydrate	55.00	49.92
Fat	4.50	20.97
Crude fiber	8.00	4.99
Ash/mineral ingredients	10.00	3.89
Vitamin ingredients	ND	0.30
Cholesterol	0	0.15
**Energy contribution** **(% total kcal)**		
Protein	25.7	17
Carbohydrate	62.8	43
Fat	11.5	40
**Total energy (kcal/100 g)**	350	467

## Data Availability

The original contributions presented in this study are included in the article. Further inquiries can be directed to the corresponding author.

## Abbreviations

The following abbreviations are used in this manuscript:

ALT	Alanine aminotransferase
ApoE	Apolipoprotein E
AST	Aspartate aminotransferase
AUC	Area under the curve
BSA	Bovine serum albumin
cDNA	Complementary DNA
CRD	Carbohydrate recognition domain
CTL	Control
DAB	3,3′-Diaminobenzidine
DAG	Diacylglycerol
ECL	Enhanced chemiluminescence
ELISA	Enzyme-linked immunosorbent assay
F4/80	Murine macrophage marker
Gal-3	Galectin-3
GTT	Glucose tolerance test
H&E	Hematoxylin and eosin
HOMA-β	Homeostasis Model Assessment of β-cell function
HOMA-IR	Homeostasis Model Assessment of Insulin Resistance
HSC	Hepatic stellate cell
i.p.	Intraperitoneal
JNK	c-Jun N-terminal kinase
MASLD	Metabolic dysfunction-associated steatotic liver disease
MASH	Metabolic dysfunction-associated steatohepatitis
MCP	Modified citrus pectin
OLTT	Oral lipid tolerance test
ORO	Oil Red O
PBS	Phosphate-buffered saline
PCR	Polymerase chain reaction
p-AKT2	Phosphorylated AKT2
p-JNK	Phosphorylated c-Jun N-terminal kinase
PVDF	Polyvinylidene difluoride
RIPA	Radioimmunoprecipitation assay buffer
RNA	Ribonucleic acid
RT-qPCR	Reverse transcription quantitative polymerase chain reaction
SD	Standard diet
SDS-PAGE	Sodium dodecyl sulfate–polyacrylamide gel electrophoresis
SEM	Standard error of the mean
TGF-β	Transforming growth factor beta
TRIzol	TRIzol reagent
WD	Western diet
